# Structure-Function Relationships of the Follicle-Stimulating Hormone Receptor

**DOI:** 10.3389/fendo.2018.00707

**Published:** 2018-11-29

**Authors:** Alfredo Ulloa-Aguirre, Teresa Zariñán, Eduardo Jardón-Valadez, Rubén Gutiérrez-Sagal, James A. Dias

**Affiliations:** ^1^Red de Apoyo a la Investigación, Universidad Nacional Autónoma de México and Instituto Nacional de Ciencias Médicas y Nutrición Salvador Zubirán, Mexico City, Mexico; ^2^Departamento de Ciencias Ambientales, Universidad Autónoma Metropolitana Unidad Lerma, Lerma, Mexico; ^3^Department of Biomedical Sciences, School of Public Health, University at Albany, Albany, NY, United States

**Keywords:** follicle-stimulating hormone receptor (FSHR), follitropin receptor, structure, G protein-coupled receptor (GPCR), glycoprotein hormone receptors

## Abstract

The follicle-stimulating hormone receptor (FSHR) plays a crucial role in reproduction. This structurally complex receptor is a member of the G-protein coupled receptor (GPCR) superfamily of membrane receptors. As with the other structurally similar glycoprotein hormone receptors (the thyroid-stimulating hormone and luteinizing hormone-chorionic gonadotropin hormone receptors), the FSHR is characterized by an extensive extracellular domain, where binding to FSH occurs, linked to the signal specificity subdomain or hinge region. This region is involved in ligand-stimulated receptor activation whereas the seven transmembrane domain is associated with receptor activation and transmission of the activation process to the intracellular loops comprised of amino acid sequences, which predicate coupling to effectors, interaction with adapter proteins, and triggering of downstream intracellular signaling. In this review, we describe the most important structural features of the FSHR intimately involved in regulation of FSHR function, including trafficking, dimerization, and oligomerization, ligand binding, agonist-stimulated activation, and signal transduction.

## Introduction

**Figure 1:**
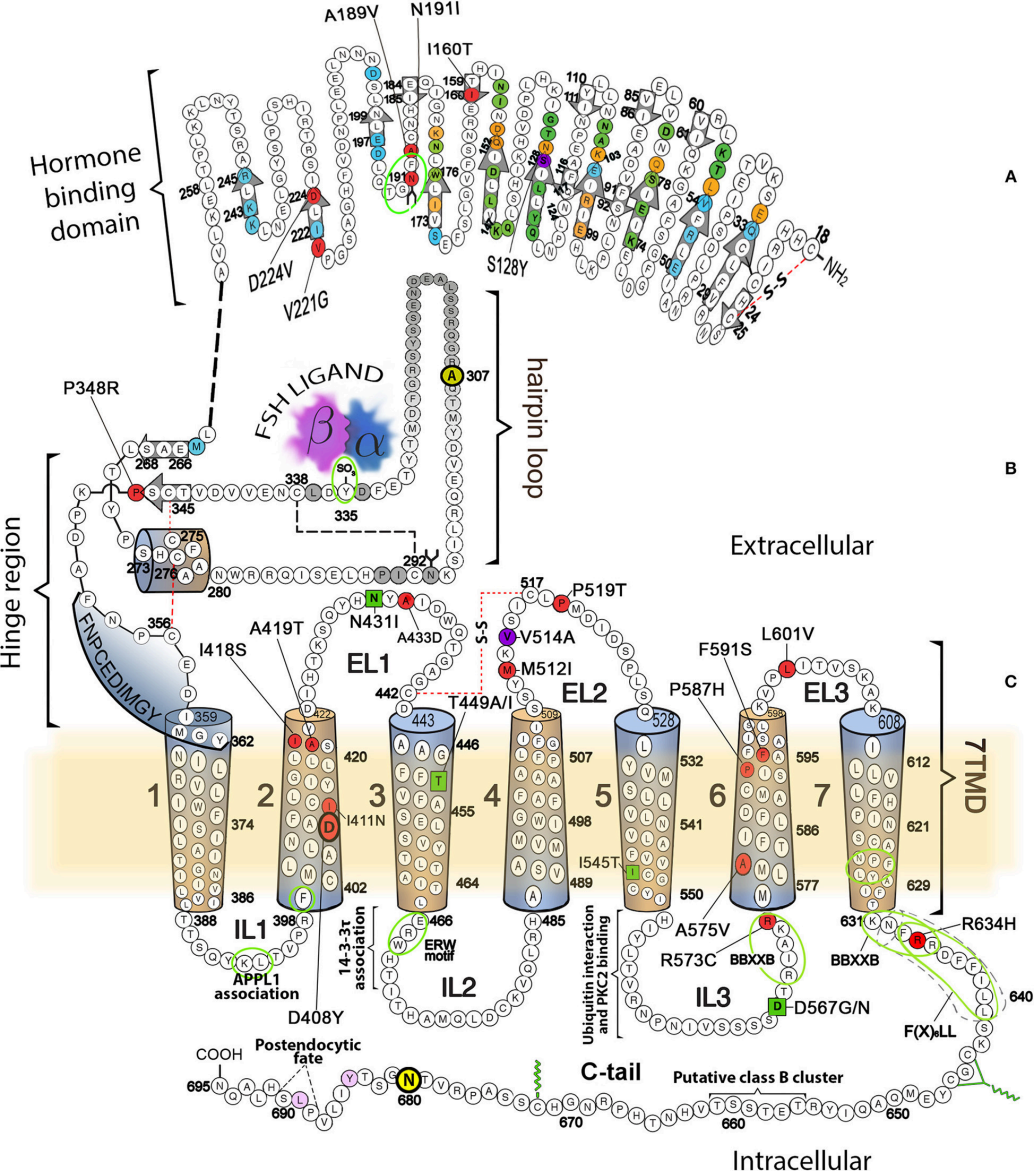
Schematic representation of the FSHR, showing its amino acid sequence and domains involved in different receptor functions, including binding to agonist, activation, and signal transduction. **(A)** Hormone specific binding domain. Residues buried in the FSH/FSHR interface and located in the high affinity-binding site are colored circles (green, binding to FSH α-subunit only; blue, binding to FSH β-subunit only; orange, residues that interact with both FSH subunits). Beta strands located in the concave (corresponding to the leucine-rich repeats) or convex surface of ECD are indicated by the colorless arrows. Mutations in this domain leading to promiscuous ligand binding are depicted in magenta (S128Y), whereas mutations in residues leading to loss-of-function are colored in red. The majority of these mutations provoke defects in receptor trafficking. **(B)** Hinge region with the sulfated tyrosine (in position 335) involved in ligand-provoked binding to the FSH subunits is indicated by the green oval. **(C)** 7TMD with the α helices represented as cylinders. The location of naturally occurring loss-of-function mutations are shown as red-colored circles, while the gain-of-function mutations are represented by green squares. The mutation at V514 (magenta circle at the EL2), led to increased plasma membrane expression of the receptor and OHSS at low FSH doses [reviewed in (4)]. Also indicated are sequences and residues located in the cytoplasmic side involved in association of the receptor with interacting proteins, receptor activation, upward trafficking, internalization, and post-endocytic fate. For details, see the text.

The glycoprotein hormone (GPH) receptors (GPHR), are members of the highly conserved Class A subfamily (or rhodopsin-like family) of the G protein-coupled receptor (GPCR) superfamily (1–5). GPCRs are 7-transmembrane-helix protein molecules that transmit intracellular effects through activating intracellular signaling mediated by members of the guanine-nucleotide-binding signal-transducing proteins (G proteins); they are characterized by a single polypeptide chain that traverses the lipid bilayer of the plasma membrane seven times, forming characteristic transmembrane α-helices linked by alternating extracellular and intracellular sequences or loops, with an extracellular amino-terminus end and an intracellular carboxyl-terminal tail (C-tail) of variable lengths. In the case of GPHRs, common features include a large amino-terminal extracellular domain (ECD), where recognition and binding of their cognate ligands, follicle-stimulating hormone or follitropin (FSH), luteinizing hormone (LH), and thyroid-stimulating hormone (TSH) occur (6). This domain contains a central structural motif of imperfect leucine-rich repeats [12 in the FSH receptor (FSHR), 9 in the luteinizing hormone/chorionic gonadotropin receptor (LHCGR) and 11 in the TSH receptor (TSHR) (7–9)] that is shared with several cell surface plasma membrane receptors. The leucine-rich repeats motif comprises a surface that is involved in selectivity for ligands and specific protein-protein interactions, and is formed by successive repeating units (β-strand and α-helix) that collectively predispose the ECD to adopt a horse shoe-shaped tertiary structure (see Figure 1A in the schematic representation of the FSHR, a prototypical member of the GPHR family) (7, 10). At the COOH-terminal end of the large ECD resides the “hinge” region, which links the leucine-rich repeat (LRR) ECD with the serpentine, seven-transmembrane α-helical domains (7TMD) and that plays a critical role of signaling functionality of the receptor (7) (Figure 1B). The hinge region of all GPHRs is involved not only in high affinity binding of the ligand but in also receptor activation, intramolecular signal transduction and silencing of basal activity in the absence of ligand (9).

The *FSHR* is about 190 Kb long and is located on chromosome 2p21–p16 (11); its coding region comprises 10 exons, each varying in size from 69 to 1,234 bp, and 9 introns with sizes 108 to 15 kb. Exons 1–9 of the receptor gene encode the large ECD, including the hinge region, whereas exon 10 encodes the COOH-terminal end of the hinge region, the 7TMD (which contains 3 extracellular loops and 3 intracellular loops) and the intracellular C-tail (3, 11). The human FSHR (hereafter abbreviated as only FSHR) protein is composed of 695 amino acid residues; the first set of 17 amino acids encodes the signal sequence, which after cleavage results in a predicted cell surface plasma membrane (PM)-expressed, mature FSHR of 678 amino acid residues exhibiting an approximate molecular weight of 75 kDa as predicted from its cDNA sequence (12). However, further cleavage of the FSHR occurs at the C-tail, but the exact location of this cleavage has yet to be determined (13). Three of four potential N-linked glycosylation sites yields receptor forms with molecular weights (as determined by gel electrophoresis) of ~80 to ~87 kDa for the mature receptor (14). A high degree sequence homology is present in both the FSHR and its closely related LHCGR. In fact, their sequence homology is ~46% in the ECD and ~72% in the 7TMD (12, 15). Of the three domains of the gonadotropin receptors, the intracellular sequences, which include the intervening loops and the C-tail, present the lowest sequence homology (~27% identity), except the NH_2_-ends of the carboxyl-termini, which have cysteine residues for palmitoylation and the primary sequence motif [F(*X*)_6_LL] that is involved in intracellular trafficking from the endoplasmic reticulum to the PM (16–18). Both of these structural features are quite common in the rhodopsin-like GPCR Class and likely play a role in signaling specificity particularly when two members of the same family (FSHR and LHR) are coexpressed in the same cell (granulosa cell).

Gonadotropins and their receptors play an essential role in reproduction. In the ovary, FSHR is predominantly expressed in granulosa cells of developing follicles, where the FSH-activated receptor triggers activation of a complex signaling network that promotes follicle growth and maturation, and induces in the granulosa cells the necessary enzymes for converting the androgens provided by the theca cells under the LH stimulus to estrogens (19). In the testis, the Sertoli cells lining the seminiferous tubules are the targets of FSH action, where the gonadotropin promotes their growth and maturation and, together with testosterone produced by LH-stimulated Leydig cells, initiates, and supports high quality spermatogenesis (20, 21). Interestingly, a recent study in transgenic mice showed that a constitutively active mutant (CAM) FSHR may support normal spermatogenesis alone in the absence of androgens (22). Whether this finding in mice is relevant in humans remains an open question.

In recent years there have been reports of FSHR detected in other than the canonical gonadal tissues. Extragonadal FSHRs, which include bone (23, 24), monocytes (25, 26), different sites of the female reproductive tract and the developing placenta (27), endothelial cells from umbilical vein (28) and blood vessels from malignant tumors and metastases (29–31), and the liver (32), have been identified employing different detection approaches, mainly immunohistochemistry and more recently *in vitro* and *in vivo* imaging of FSH-conjugated NIRII-fluorophore (33). It has been proposed that these extragonadal FSHRs might play a role in diverse physiological processes, mainly related with osteoclast-mediated bone resorption and angiogenesis (34–40). However, expression of FSHRs in some extragonadal tissues has been recently questioned (41). Regarding their structure-function relationship, it is interesting to note that the FSHRs mRNA transcripts identified in human monocytes and osteoclasts apparently correspond to receptor isoforms or variants resulting from differential splicing that do not transduce signals in response to FSH *via* the canonical G_s_ protein pathway (26) but rather, probably, through G_i2_ which in turn triggers MEK/Erk, NF-kB, and Akt activation leading to increased osteoclast formation (23).

More recently, Liu and colleagues (42) showed that immunoneutralization of circulating FSH levels via administration of either a polyclonal or monoclonal anti-FSHβ antibody to mice, not only led to attenuation in bone loss in ovariectomized animals but also prevented adipose tissue accumulation and parallely enhanced brown adipose tissue and thermogenesis, probably by blocking the inhibition promoted by FSH on uncoupling protein 1 (Ucp1) expression, a regulator of white fat beiging and thermogenesis (43). Given the physiological and therapeutic implications of extragonadal FSHRs, more studies, particularly in humans, are warranted to confirm that extragonadal FSHRs are expressed at sufficient densities to evoke significant biological effects particularly when exposed to increased FSH levels, as those present during the peri- and postmenopause.

The FSHR protein includes a number of specific primary sequences involved in many of the functions of the receptor. These sequences are involved in outward trafficking from its site of synthesis (the endoplasmic reticulum; ER) to the PM (upward trafficking), agonist binding and activation, signal transduction, desensitization and internalization, and degradation or recycling (downward trafficking). Alterations in any of these primary sequences by gene mutations or due to single nucleotide polymorphisms (SNPs), may potentially result in abnormal function of the receptor protein and eventually to disease.

## Domains and motifs involved in fshr upward trafficking

The endoplasmic reticulum (ER) is the cell organelle where the life cycle of GPCRs begins; here, the newly synthesized peptide sequence is translocated, folded into secondary and tertiary structures via disulfide bonds formation and assembled into quaternary complexes. Properly folded receptors are then exported to the ER-Golgi intermediate complex and then to the Golgi apparatus and trans-Golgi network; here, processing is completed, and the receptor proteins are ready to complete their outward trafficking to the PM and become exposed to cognate ligands (44, 45). Similar to other GPCRs, if the FSHR is not correctly folded the quality control surveillance of the proteosome removes the misfolded receptor. If properly folded, in the ER FSHR continues its transit to the Golgi and the PM (46). N-linked glycosylation (as well as disulfide bond formation) is a frequent feature of GPCRs that occurs during biosynthesis and facilitates folding of protein precursors by increasing their solubility, protecting from detrimental non-productive protein-protein interactions and stabilizing protein conformation (47). Glycosylation plays a crucial role in folding, maturation, and intracellular trafficking of the receptors from the ER to the PM (48). As mentioned above, the ECD of the FSHR contains four potential N-linked glycosylation sites (sequence N*X*S/T, where *X* is any amino acid except proline) at positions 191, 199, 293, and 318 (12). However, the crystal structure of the FSHR ECD at residues 25 to 250 in complex with FSH (7, 49) (see below) has provided positive evidence for glycosylation at only one of these sites. That structure revealed that carbohydrate is attached at residue N191, which protrudes into solvent, while no incorporation of carbohydrate complex occurs at residue N199, which projects from the flat β-sheet into the hormone-receptor binding interface and if present would prevent hormone binding, as might be predicted by the FSH-FSHR ECD crystal structure (14). Information is lacking on FSHR glycosylation at residues 293 and 318, albeit some studies suggest that it might occur at two of the three (at positions 191, 199, 293) N-linked glycosylation consensus sequences (50) (Figure 1A). Naturally occurring mutations at the ECD of the FSHR (51, 52) near or at putative glycosylation sites are deleterious, emphasizing the important role of glycosylation on receptor targeting to the cell surface and insertion into the PM. In fact, the A189V, and N191I naturally occurring FSHR mutations lead to a profound defect in targeting the receptor protein to the PM, confirming the role of the conserved 189AFNGT193 motif (which hosts one glycosylation site) in FSHR trafficking. Nevertheless, it is not known whether the A189V mutant FSHR is glycosylated at position N191, given that V189 as well as I191 may potentially impair proper receptor LRR formation, particularly its α-helical portion, and hence receptor trafficking.

On the other hand, mutagenesis, and biochemical studies suggest that in the rat FSHR glycosylation is present at two glycosylation consensus sequences and that disruption of either of these two glycosylation sites (N191 or N293) does not apparently affect receptor folding and trafficking to the PM (50). The authors interpretation of this finding is that in this rodent species, at least one glycosylation site at the ECD is needed for FSHR folding and efficient trafficking to the PM (50). Abscence of glycosylation of the mature rat FSHR does not impact on binding or affinity, albeit glycans appear to be important structures for the maturation of the newly synthesized receptor helping on folding, conformational stability, and correct routing to the plasma membrane.

Mutations at the amino-terminal end of the ECD also affects cell surface residency of the FSHR. In this region, alanine scanning mutagenesis identified two regions comprising amino acid residues V9-L31 and E39-N47 which are apparently important for receptor trafficking (53, 54). Mutations in several amino acid residues, specifically at F30, I40, D43, L44, R46, and N47 significantly decreased cell surface PM expression due to failure for proper trafficking (54). Although mutations at these sites might impair glycosylation of the receptor, the abnormal trafficking was more likely due to abnormal NH_2_-terminal folding and trapping FSHR intermediates by surveillance mechanisms that incidentally may interfere with appropriate glycosylation processing in the ER-Golgi.

In addition to the above described 189AFNGT193 motif in the FSHR, where mutations influence upward trafficking of gonadotropin receptors, other sequence motifs located in intracellular domains seem to be involved in the exit of these and other GPCRs from the ER and the Golgi. Among these export motifs is the F(*X*)_6_LL (where *X* is any amino acid) sequence described by Duvernay and colleagues (16, 55) located between residues 633 and 641 in the FSHR (Figure 1C). The C-tail sequence of the FSHR also contains the minimal BB*XX*B motif reversed (B*XX*BB, where B represents a basic amino acid and *X* any other amino acid) in its juxtamembrane region (residues 631KNFRR635) (56); the last arginine residues of this latter motif (at positions 634 and 635) and the preceding F633 also are included within the NH_2_-terminal end of the F(*X*)_6_LL sequence, and hence substitutions in these residues impaired trafficking and PM expression of the receptor (56, 57). The intracellular loop (IL) 3 of the FSHR also contains this B*XX*BB motif (residues 569RIAKR573) and either deletion or replacement of its basic residues with alanine also impairs PM expression of the receptor (56, 58).

There are other naturally-occurring mutations that affect trafficking of the FSHR besides those at exon 7 already described, as well as those that impact on ECD glycosylation. These have been identified by virtue of their causal relationship to intracellular retention of FSHR and include the I160T and D224V mutations (at exons 6 and 9, respectively) at the ECD (59, 60), D408Y at the TMD2 (61), and P519T at the extracelullar loop (EL) 2 (62) (Figure 1 red-filled circles). There have been few studies of the molecular physiopathogenesis leading to impaired upward trafficking of these FSHR mutants. The Pro519Thr mutation in the middle of the EL2 results in complete failure of FSHR to bind FSH and incompetence for triggering intracellular signaling. The loss of a proline residue at this position may potentially provoke a severe conformational flexibility that leads to misfolding and intracellular trapping of the mutant receptor. The peptide backbone of proline, which is constrained in a ring structure, is associated with a forced turn in the protein sequence, which is lost when the less constraining threonine is present instead. Thus, it is possible that the abrupt turn at the middle of the EL2 [where the highly conserved motif KVSIC*X***P**MDV/T/I (residues 513–522 in the FSHR) present in all three glycoprotein hormone receptors is located], may be an obligatory requisite for both signal transduction activity and proper routing of the receptor to the PM membrane (62). The remaining mutations (at positions 160, 224, and 408) also occur at highly conserved residues or sequences across species (12), supporting their importance on FSHR function, at least on its proper intracellular routing to the PM.

**Figure 2:**
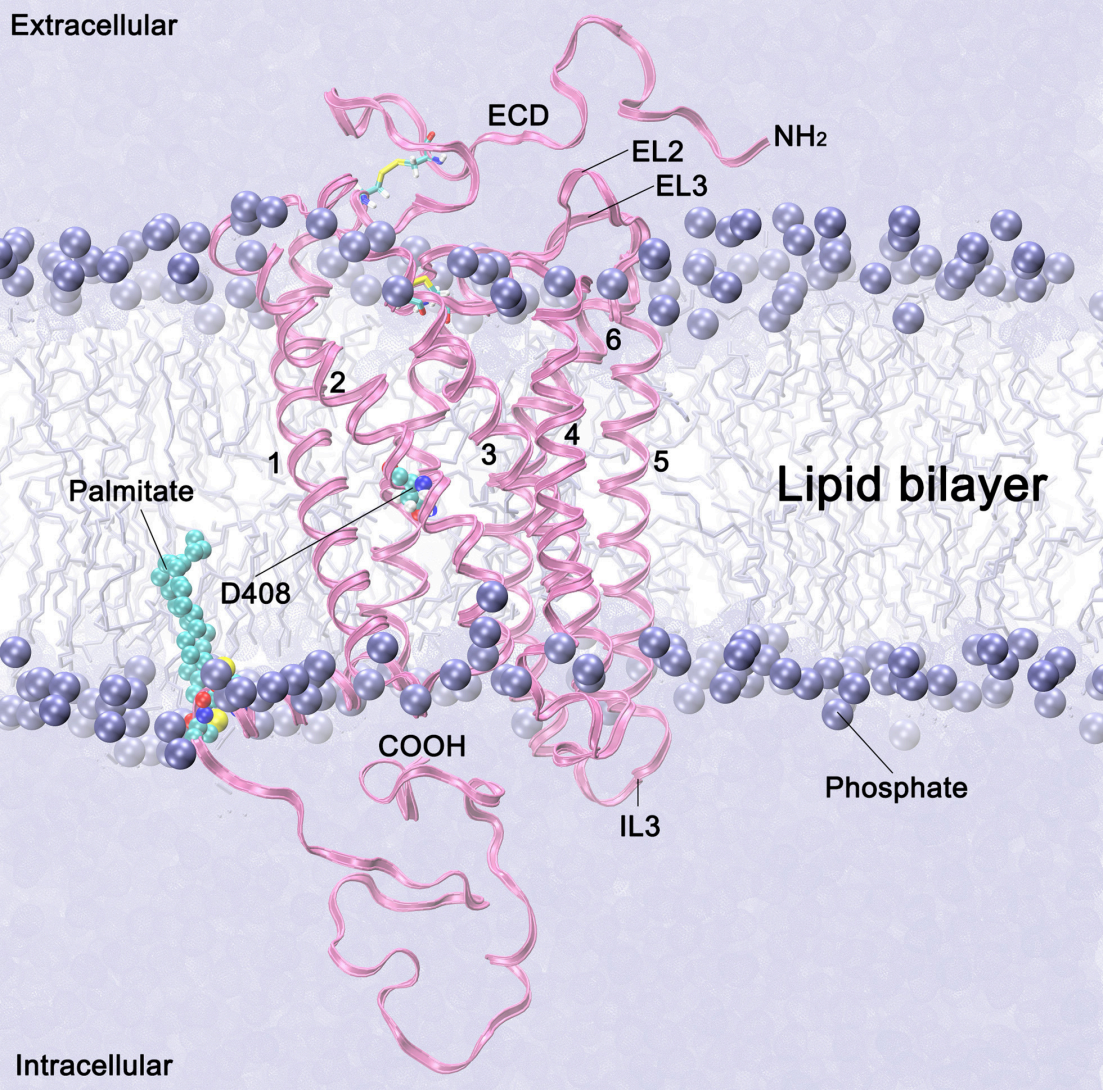
Follicle stimulating hormone receptor (magenta ribbons) in a lipid membrane bilayer (violet spheres and sticks). The 7TMD domains are identified with numbers 1–7. Extracellular loops 2 and 3, and the intracellular loop 3, are labeled as EL2, EL3 and IL3, respectively. The NH_2_ terminus with a fragment of the ectodomain (ECD) (starting at residue 317) is depicted in the extracellular side. Palmitoylated cysteine residues anchored in the membrane are depicted in cyan spheres. The lipid heads are represented by the phosphorous atoms, which are depicted as violet spheres, and the lipid tails are represented as free-drawn vertical lines in the background. Water molecules at the intra- and extraccelular sides are depicted as a continum solvent in violent.

**Figure 3:**
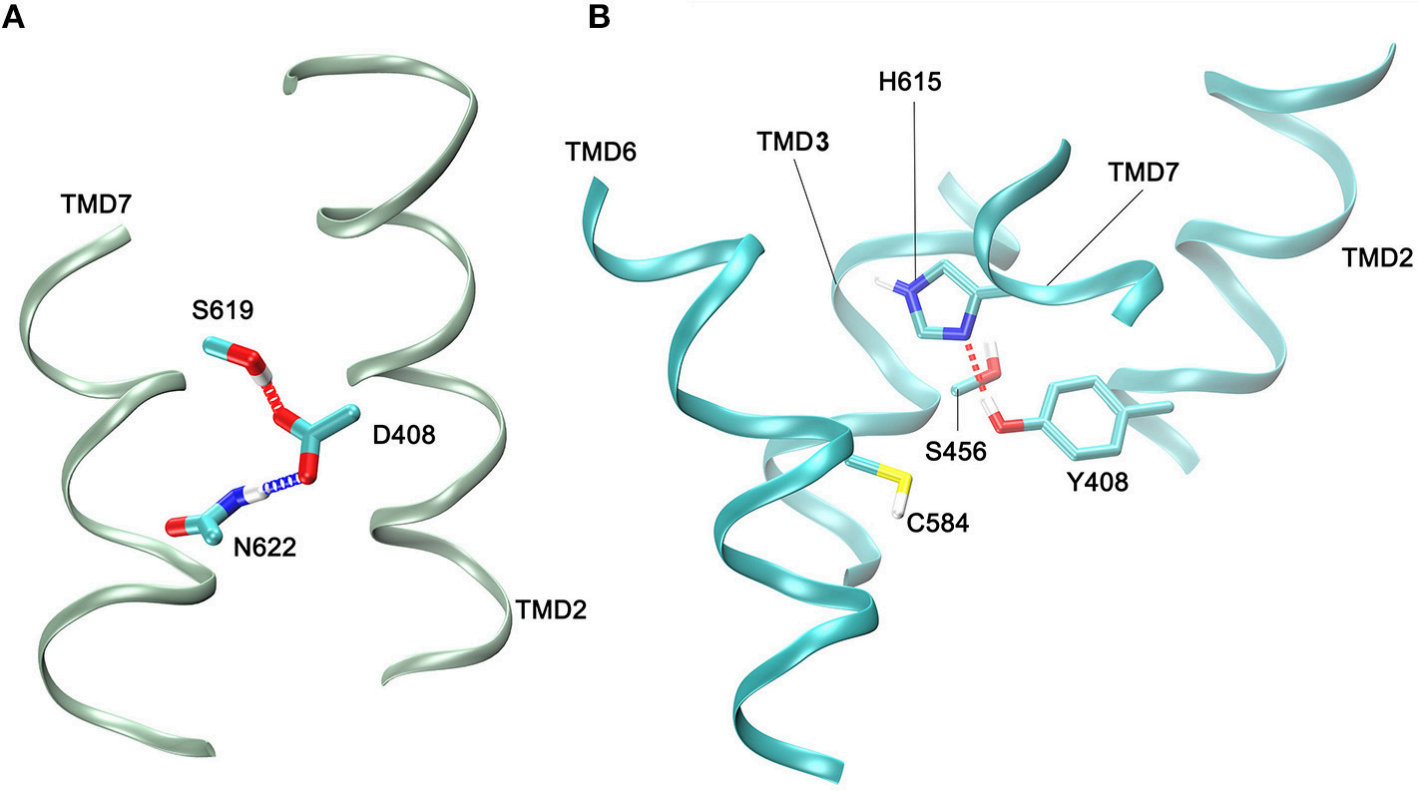
Contacts between side chain atoms of residues at helix 2 (TMD2) and residues at helices 3 (TMD3), 6 (TMD6), and 7 (TMD7). **(A)** Side chain interactions in the WT FSHR with the carboxyl group of D408 forming hydrogen bonds with S619 and N622. **(B)** Side chain interactions of Y408 at TMD helix 2 and residues at TMD helix 3, helix 6, and helix 7. A hydrogen bond between Y408 and H615 is shown; C584 is depicted since it is close neighbor of Y408 within a 3.5 Å cut-off. Side chains are depicted as sticks, and the color code is: carbon,cyan; oxigen,red; hydrogen, white; sulfur,yellow; and nitrogen, blue. Only small fragments of the helical regions are depicted (green or cyan ribons for the WT and 408 mutant receptors, respectively).

**Figure 4:**
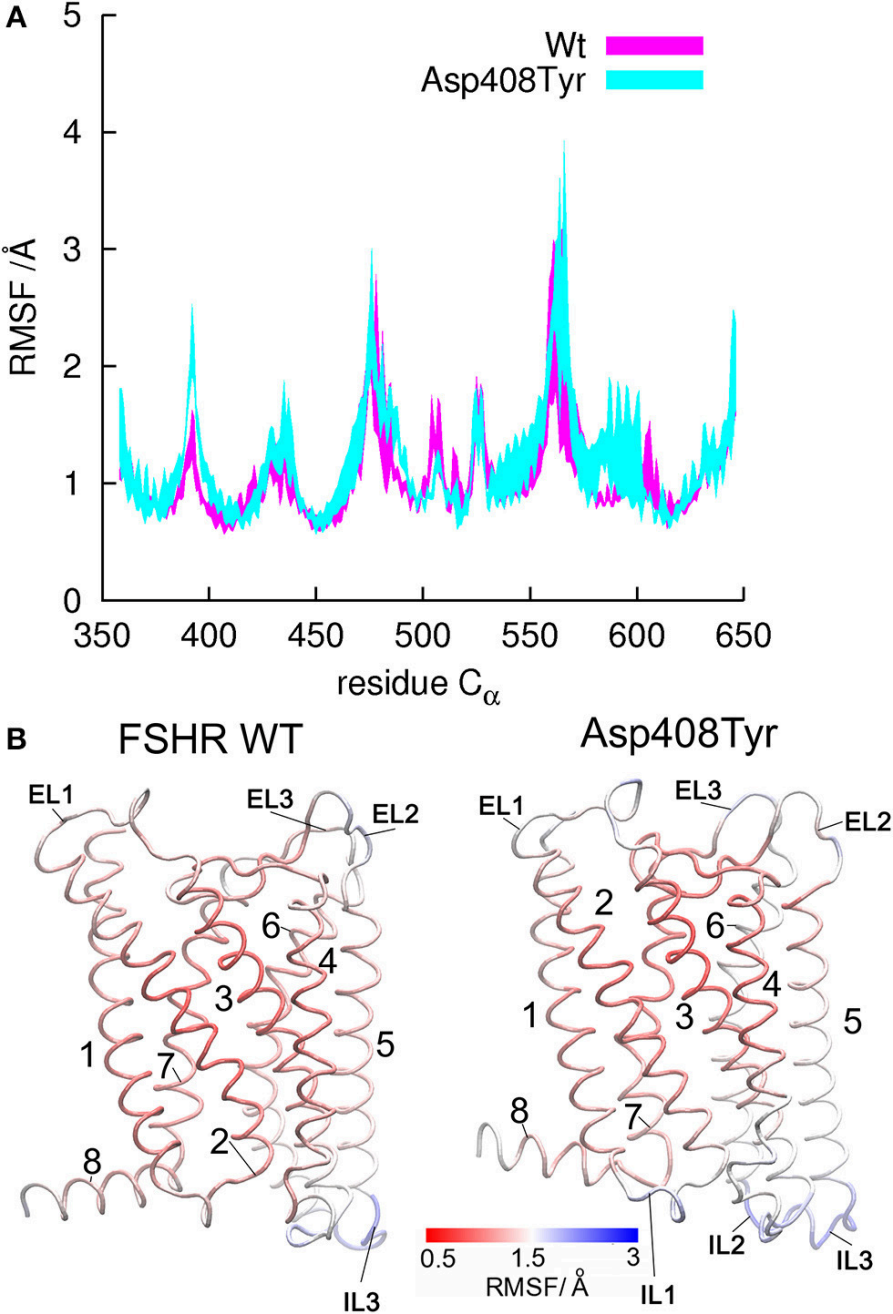
Root mean square fluctiations (RMSF) for α-carbon atoms of the WT FSHR and the mutant D408Y. **(A)** Fluctuations calculated for residues in the tansmembrane domain from Y362 to C646. Helical regions display lower RMSF values since they are rather rigid within the bilayer hydrophobic core, whereas larger fluctuations represent flexible regions such as the loops. **(B)** Structures of the WT FSHR and the D408Y mutant colored according to the RMSF values, with rigid regions in red and flexible regions in blue. Flexibility seems to increase from helix 5 to helix 8 in the mutant receptor, since larger RMSF values were yield by the mutant than by the WT FSHR.

The above mentioned FSHR D408Y mutation represents an interesting paradigm to explore the molecular mechanisms subserving misfolding and impaired intracellular trafficking of mutant FSHR to the PM. Potential alterations in the secondary structure of the D408Y mutant receptor have been proposed using template-based modeling techniques. Bramble et al. (61) compared a model of the WT FSHR to a model of FSHR containing the D408Y mutation using the RaptorX software (63). The exercise detected a distorted helical structure upstream at the site of the mutation at the 7TMD helix 2; this observation was corroborated by a calculated decreased in the helicity score of the 400 to 410 region using ExPASy secondary structure predictor (61). A caveat should be noted, however, that template-based-modeling relies on known structures of proteins (templates) that display sequence homology with the unknown protein [by homology modeling or fold recognition of individual amino acids in the context of all known structures (protein threading)]. Therefore, the accuracy of prediction of protein structure using template-based modeling of membrane proteins will be limited by the fact that there are not many solved structures of GPCR TM domains. This will likely resolve in the near future as the use of cryo-electron microscopy becomes more accessible to scientists studying GPCRs (64), which undoubtedly will transform further understanding of how GPCRs function. In the absence of such advances and complementary to this new resource, alternative approaches, such as molecular dynamics (MD) simulations, had emerged (65). All-atom MD simulations provides atomistic grounds for understanding membrane folding processes, protein-lipid affinity, and protein conformational changes, among other important phenomena for studying membrane proteins physiology in an aqueous environment. For example, in the case of the D408Y FSHR mutant, all atom MD simulations performed for a period of 20 ns within a lipid bilater environment of polyunsaturated lipids predicted that mutations at residue 408 would only affect *very slightly* the secondary structure. This is because the H-bonds stabilizing the helical domains are located in the hydrophobic core of the bilayer, where electrostatic interactions are enhanced due to the non-polar environment of the lipid hydrocarbon tails. However, contacts between TMD2 and TMD7 are indeed disrupted upon replacement of aspartic acid with tyrosine at position 408 (Figure 2). Here Y408 made contacts (with S456 at TMD3, C584 at TMD6, and H615 at TM7) not observed in the WT receptor (Figure 3). This indicated that replacements at position 408 may severely impact on the conformational dynamics of the receptor and thereby promote distinct fluctuations throughout to the whole receptor structure (Figure 4), which may potentially lead to *misfolding* and *retention* of the mutant receptor within the cell by the quality control system of the cell.

### Dimerization and upward trafficking

Association between GPCRs, either in the form of dimers or oligomers, plays a pivotal role in GPCR function, influencing intracellular trafficking, ligand binding, and signaling regulation (66–68). In the case of the FSHR, the receptor self-associates early during receptor biosynthesis, and using both biochemical and super-resolution imaging approaches evidence supports the quaternary association at the PM as both monomers and higher order structures (dimers and oligomers) (13, 69, 70). Nevertheless, whether association of FSHRs in the ER is an *obligatory* pre-requisite for trafficking to the PM, as with other GPCRs (71–77), is an open question. Although biochemical studies have found that both the ECD and TMD contribute to early FSHR association, the sites of interaction(s) remain to be identified. In fact, in one study (69), mutations in TMD helix 1 and/or 4, which have previously been suggested to be involved in dimerization of the α1β-adrenergic receptor, dopamine D2 receptor, and CCR5 (78–80), failed to alter the propensity of the FSHR to associate. Nevertheless, some domains potentially involved in intracellular FSHR-FSHR interactions have been identified employing short interfering sequences specific for particular TMDs and the C-tail (57). That study suggested that association of FSHRs may occur via multiple contact sites at the 7TMD, including helices 5, 6, and 7, and the C-tail. Although in how this FSHR-FSHR interaction might influence upward traffic of the FSHR to the PM has not yet been particularly addressed, the same study also demonstrated that heterozygous mutations causing misrouting of the receptor led to defective upward intracellular trafficking and interfered with proper maturation of the WT, functional FSHR (57). The more recent crystal structure of the FSHR ECD, which included the entire 350 amino acid of the ECD, demonstrated an additional mode of association of hormone with the ECD that includes the hinge region of the receptor (Figure 1B) and represented a trimeric receptor structure (14, 81). This latter observation will be an important platform for defining the number of FSH molecules hosted by the receptor but whose formation during the biosynthetic process and role in receptor trafficking has not been yet documented. In this vein it is possible that association of FSHR receptors as dimers or trimers may facilitate coupling the receptor to several and distinct G proteins and adaptors. In fact, a recent study has shown that heteromers of adenosine A2A receptor and dopamine D2 receptor homodimers associated to distinct G proteins, may modulate signal transduction selectivity through different molecular interactions with effectors (82).

Heterodimerization of FSHR with the closely related LHCGR, has been studied employing different experimental approaches (57, 70, 83, 84). However, it is not yet known whether such hetero-association also occurs early during biosynthesis, as demonstrated for FSHR homodimers, or later, when the individual receptors are already at the cell surface PM. In any case, the presence of FSHR-LHCGR heterodimers appears to convey important physiological implications, particularly during follicular maturation, as it may prevent premature luteinization of the follicle or ovarian hyperstimulation, according with the level of expression of each receptor (83). As in the case of association between FSHRs, it is still unknown which are the potential contact sites of interaction between these receptors, although experiments using mutant FSHRs coexpressed with the WT LHCGR suggest that this hetero-association may also occur via multiple inter-TMD contacts (57).

## FSHR domains involved in ligand binding and receptor activation

### The extracellular domain (ECD) and ligand binding

As described above and shown in Figure 1A, the mature, PM expressed FSHR exhibits a large ectodomain, where recognition and binding of its cognate ligand occurs (14). The current dogma is that in the FSHR ECD resides both the binding site for agonist and the region essential for ligand-provoked triggering of receptor activation. The first reported structure of the FSH complexed with the extracellular-hormone binding domain of the FSHR (FSHR_HB_) (49) documented the important structural relationship between FSH and FSHR. However, the expressed protein used for crystalization did not include the signal specificity subdomain or hinge region, which had been considered as a separate structure participating on FSHR activation (85–87). This groundbreaking structure showed for the first time that FSH binds to FSHR_HB_ like a “handclasp” and that most β-strands in the inner surface are involved in ligand binding (Figure 1A). Moreover, extensive previous mutagenesis and biochemical analyses of FSH mutants provided an immediate validation of the dogma that both non-covalently linked α- and β-subunits (present in all glycoprotein hormones) are involved in specific binding to the receptor. Importantly, this structure also demonstrated that carbohydrates *are not* actually involved in the formation of the binding interface of the FSH–FSHR_HB_ structure, but are rather sequestered to the periphery of the complex (88, 89). This observation would argue against the notion that pharmacodynamics of FSH biosimilars may vary depending on their carbohydrate composition. The second and subsequent crystal structure of the FSH-FSHR complex shed additional light on this topic while suggesting even more complicated structure-function correlates to consider.

**Figure 5:**
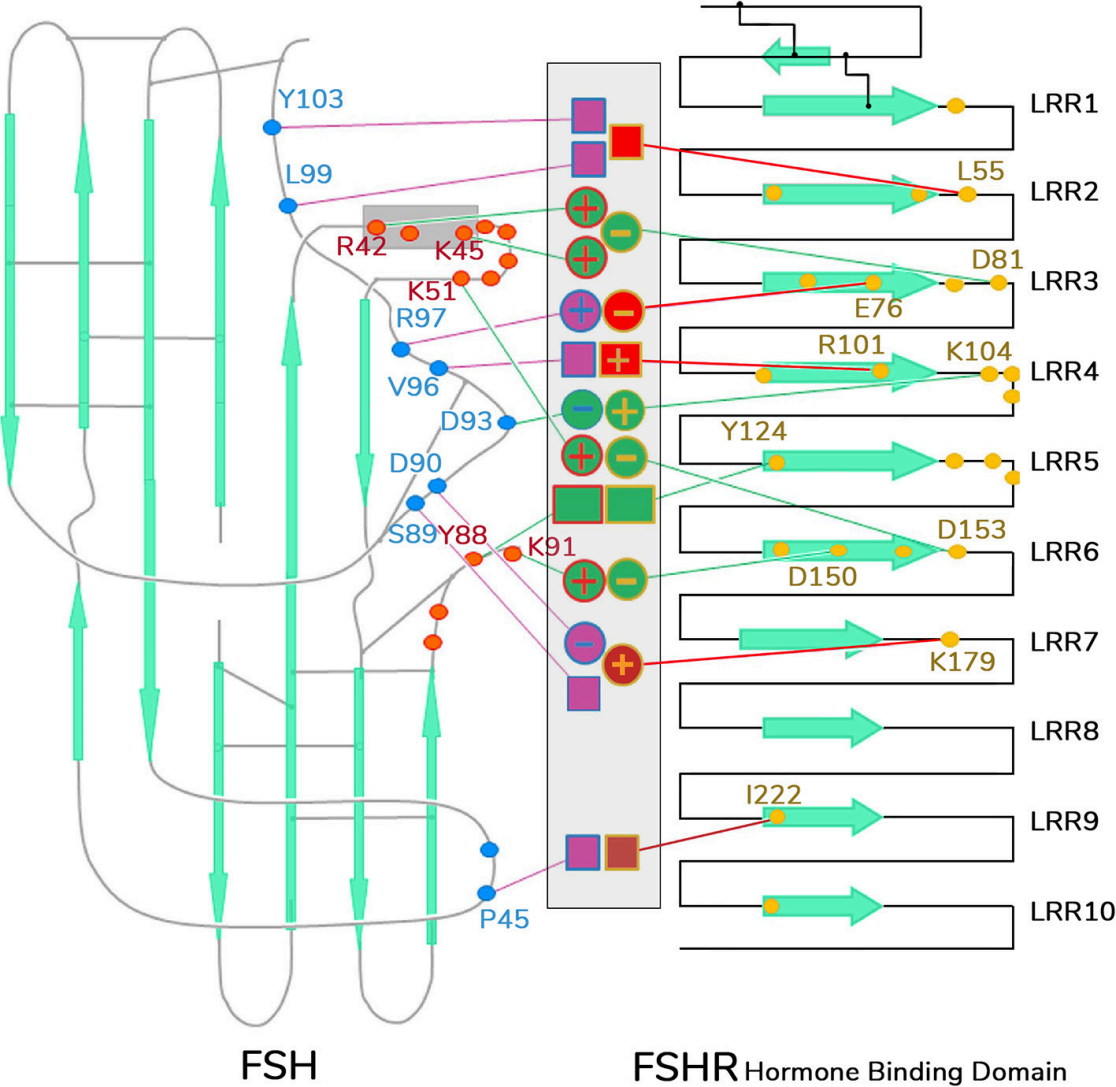
Schematic representation of detailed interaction of FSH and FSHR interface. Contacting residues from FSHR hormone binding domain are shown as yellow dots, those from FSHα as red dots, and FSHβ as blue dots. The middle area indicates the specific side-chain interactions between FSHR and its ligand. Interactions that contribute to common affinities among all the GPH–GPHR family members are shown as green-filled circles (for charge–charge interactions) or boxes (for non-charged atomic contacts), and they are connected by green lines toward the yellow dots in FSHR or red or blue dots in FSH α- or β-subunits, respectively. Interactions involved in specificity are shown as purple- or red-filled circles or boxes connected by lines of the same color to the dotted residues in the receptor and ligand. LRR, leucine-rich repeats.

The second crystal structure of FSH bound with the entire FSHR ECD reported by Jiang and colleagues (7) includes the hinge region (FSHR_ED_). That structure described in more detail the role of the glycoprotein hormones receptors ECD not only in ligand binding but also on receptor activation. Accordingly, this structure predicts that FSH is initially recruited by the previously described FSHR_HB_ through high-affinity interactions between the gonadotropin and the concave surface of leucine-rich repeats (Figure 1A, gray arrows within the amino acid sequence of the ECD) 1–8. However, the interface between the FSH and the FSHR ECD is broader than that previously identified in the Fan and Hendrickson FSH-FSHR_HB_ structure (49) due to the presence of secondary interaction sites (shown also in Figure 1A). According to this newer structure, binding of FSH to the FSHR hormone binding domain provokes conformational alterations in the L2β loop (residues V38β-Q48β) of FSH leading to interactions between amino acid residues in the L2β loop and LRRs 8 and 9, as well as to interactions of FSHR residues located in the hinge region with residues on FSH α- and β-subunits. Several residues on the FSHR determine specificity of the receptor for its ligand, including L55, E76, R101, K179, and I222, in which L55 and K179 are important to distinctly identify LH, human chorionic gonadotropin (hCG) and FSH due to their interaction with the FSHβ “seat belt,” whereas the other residues dictate specificity preventing binding to TSH (7, 14). A more detailed map of interaction between residues from FSH and the FSH_ED_ is shown in Figure 5. The FSHR ECD structure reported by Jiang and colleagues (7), identified the hinge region as an *integral* part of the ECD (Figure 1B), and confirmed previously reported biochemical data on the FSHR and TSHR (85, 90–93), underlying the role of this region in ligand-stimulated receptor activation. These and other studies (94) have also suggested that the ECD of the glycoprotein hormone receptors acts as a tethered inverse agonist. In this scenario, the ECD acts as an agonist upon ligand binding and activates the sequence 353FNPCEDIMGY362 located in the junction of the carboxyl-terminal end of the hinge region and the 7TMD helix 1, which function as an internal agonist unit (Figure 1B).

The agonist-stimulated activation mechanism of the FSHR includes a sulfated tyrosine residue at position 335 of the hinge region. Here, exposure of a pocket located in the interface of the α- and β-subunits of FSH formed upon binding of the ligand to the hormone binding domain, is the binding site for the sulfated tyrosine residue located immediately adjacent to the rigid hairpin loop (Figure 1B). The proposal is that this initial binding event is followed by lifting of the hairpin loop leading to relieving of the inhibitory effects of the loop on the 7TMD. Rotation of a fixed short helix formed by residues S273 to A279 (Figure 1B) additionally contributes to the conformational change of the hinge region that leads to receptor activation (7, 14). The fact that substitution of the S273 residue with a non-polar hydrophobic residue (isoleucine; S273I) leads to constitutive activation of the receptor, emphasizes on the importance of this helix movements on FSHR activation; this mechanism may also explain the effect of the S277I mutation on LHCGR constitutive activation (95). The disulfide bridges C275-C346 and C276-C356 play an additional role in FSHR activation through fastening the last β-strand (LRR 12) to the short helix forming a rigid body and tying this helix to the last few residues before the 7TMD helix 1 (internal agonist in Figure 1B). The movement of the hairpin loop occurring upon ligand binding presumably affects and influences the conformation of this and the remaining TMDs, thereby provoking receptor activation (see below). This structure has far-reaching impact. Given the similarity among the structures of glycoprotein hormones and glycoprotein hormone receptors, it is highly possible that all glycoprotein hormone receptors share the 2-step recognition/activation process described above. For example, mutants of glycoprotein hormone receptors created to remove this critical sulfated tyrosine, exhibit a marked loss of sensitivity to their corresponding ligands (86, 87, 96). Moreover, FSH with mutations in residues located below the sulfated tyrosine-binding pocket or at the potential exosite (αF74E and βL73E, respectively) promote signaling presumably by taking the hairpin loop up toward the top of the pocket (7).

FSHR (and TSHR as well) promiscuity for ligand specificity caused by particular mutations in the ECD (and the 7TMD as well, see below) (Figure 1) is an issue that has important implications in the clinical setting. This is because of the structural similarities among the glycoprotein hormones and their receptors and the limited number of residues in the ligand and the LRRs at the hormone-binding domain that participate in ligand-receptor interactions. For example, a ligand structurally similar to a glycoprotein hormone receptor cognate ligand could interact with and activate the receptor. This could even occur with a low affinity and without triggering detectable receptor activation under basal conditions. In this regard, replacements of key residues that presumably participate in receptor-ligand interaction may hamper ligand discrimination of the receptor and result in recognition and interaction of the mutant receptor with other than its specific ligand. In this setting, the S128Y mutation at the FSHR (Figure 1) may provoke ovarian hyperstimulation syndrome [OHSS; which may be life-threatening in its severe form (97)] associated to pregnancy due to increased responsiveness of the FSHR to high levels of hCG present during the first trimester of pregnancy (98). In this mutation, replacement of serine with tyrosine allows the FSHR to hydrogen bond αR95 at the hCG molecule, leading to receptor activation.

Since the ELs are extracellular projections of the TMDs, it was anticipated that these loops also may be involved in ligand-receptor interaction and receptor activation, particularly EL1 and EL3, which is, indeed, the case. The role of the FSHR ELs in these processes has been described in detail in a recent review (4).

### The 7TMD and receptor activation

Given that no structural data are currently available on gonadotropin receptors 7TMD, homology modeling with other GPCRs has been a very useful tool to explore the potential molecular mechanisms occurring at the 7TMD level that lead to the initial activation of FSHR by its ligand. Among a number of ligand-bound GPCR structures currently available, the following structures are important to understand the activation mechanism: a. A ligand-free form of opsin that is co-crystalized with the carboxyl-terminus of the α-subunit of G_α*t*_ (99); b. A β_2_AR bound to agonist and stabilized in the active conformation by a nanobody mimicking the G protein (100); c. Agonist-bound β_2_AR and adenosine A_2A_ receptor co-crystallized with heterotrimeric stimulatory G protein (Gα_s_−β_1_γ_2_) (101, 102); and d. The structures of four GPCRs bound to G_i_ obtained through cryo-electron microscopy (103–106). Previously described crystal structures, may be useful as a first approximation of ligand-induced activation of FSHR. However, since none of those receptors entertain a large extracellular domain for ligand binding, the common structural rearrangements noted may not translate well to the FSHR or other glycoprotein hormone receptors (107). Upon ligand binding, the extracellular portion of the 7TMD is initially affected by agonist-evoked local structural changes, including: a. A small distortion of TM helix 5; b. Relocation of TM helices 3 and 7; and c. Translation/rotation of TM helix 5 and helix 6. These movements occur concurrently with rearrangements in a cluster of conserved hydrophobic and aromatic residues (positions 3.40, 5.51, 6.44, and 6.48)^1^, that constitute a transmission switch deeper in the core of the receptor leading to rearrangement at the TMD helix 3–helix 5 interface, and formation of new non-covalent contacts at the TMD helix 5–TMD helix 6 interface (109). Several residues in this transmission switch are highly conserved among Class A GPCRs, suggesting that they are a common feature of GPCR activation of effector proteins. These local changes are translated into large-scale helix movements occurring intracellularly at the cytoplasmic side of the plasma membrane (107), yielding rearrangements of TMD helix 5 at its cytoplasmic side (110) associated with a modification of theTMD helix 5–helix 6 interface, which result in the large-scale relocation of the cytoplasmic side of TMD helix 6 (111). Consequently, a cleft required for *hosting G protein* α*-subunits* opens. Further, recent studies on receptor-G_i_ complexes suggest that a smaller displacement of the TMD helix 6 might interfere with binding of the receptor to G_s_ and allow to selectively bind G_i_ (103–106). Importantly, residues from the IL2 and the cytoplasmic end of TMD helix 3 (R3.50 of the conserved E/DRY/W sequence) participate in interaction with the G protein following activation (101, 112). As a result of receptor activation, the salt bridge between residues R3.50 and E6.30 in the inactive state is broken (99). These structural and biophysical studies indicate that agonist binding may not be solely sufficient to stabilize fully active states of the receptor and that binding of an effector protein on the cytosolic face of the receptor seems *necessary* to fully attain the active state of the receptor (113). Further, there may not be a single active state arguing that different ligands with or without allosteric modulators, can stabilize distinct conformations and give rise to diverse and distinct downstream responses (114, 115). It would follow then that CAM receptors might exist in conformations that facilitate recruitment of non-G protein effectors such as β-arrestins (116, 117), giving rise to biased signaling. Thus, from a clinical point of view understanding or determining their structure can guide development of therapeutically useful negative allosteric modulators. From a basic view, solving the structures of the constitutively active receptors will lead to additional insights about ligand-induced activation of FSHR, particularly with regard to engagement of downstream effectors.

Long-range conformational changes and rearrangements transmitted down stream the intracellular extensions of the TMD helices and associated with the ILs and C-tail of the receptor induce reorganization that allows accommodation and activation of multiple downstream effectors. The α-helices conforming the 7TMD may oscillate between multiple active conformations, which eventually determine the activation of several or distinct downstream signaling pathways and account for functional selectivity (see below). Given the structural and functional similarities among Class A GPCRs, it is highly possible that the FSHR (and other glycoprotein hormone receptors as well) may share some of recently described structural mechanisms of activation at the 7TMD exhibited by other members of this particular Class of GPCRs. Here it is important to note that in the case of rhodopsin the active conformation is not as variable as in other GPCRs with diffusible ligands, because upon light exposure rhodopsin exhibits (and, in fact, vision requires) a high switching fidelity and very fast activation dynamics than other GPCRs, which switch asynchronically during the ligand-stimulated activation process (118). In fact, a recent crystal structure of rhodopsin in complex with a mini-G_o_ protein (119) showed that the structure and active conformational state of rhodopsin bound to G_o_ is very similar to that previously observed for the rhodopsin-arrestin complex (120), implying that rhodopsin exposes the same sites to recognize its cognate G protein (G_t_) and arrestin and that fewer stable conformations in the active state exist in this receptor compared to other GPCRs (119).

The specific intermolecular interactions and nature of the conformational changes subserving stabilization of the glycoprotein hormone receptors 7TMD in different (inactive or active) conformations are not yet fully understood at atomic resolution. Yet evidence derived from combined experimental approaches (mutagenic, structural, and *in silico* strategies) as well as from *in vitro* recreation of naturally occurring inactivating and activating mutations (see below), have allowed identification of potential structural determinants and network interactions that predominate during the inactive and active conformations of these receptors (7, 121–126). Application of *in silico* and mutagenesis approaches, particularly on the LHCGR, have unveiled important information about TMD helices and particular amino acid residues involved in intra- and inter-helical non-covalent ionic interactions, network formation, and pathways that are associated with the different activation states of the gonadotropin receptors. In this regard, almost all conserved amino acid residues in the majority of the LHCGR helices participate in the formation of intramolecular networks in either inactive and/or active states. Moreover, highly conserved and non-conserved residues form ionic inter-helix network pathways that connect the extracellular and intracellular components of this receptor during different conformational states. Finally, salt bridging of R464 (R467 in the FSHR) at the ERW highly conserved motif located at the COOH-end of the TM3 (Figure 1C) with E463 (FSHR E466) and D564 (FSHR D567) (at the IL3-TMD helix 6 junction) represents a key network important for stabilization of the *inactive* conformation of the receptor (122, 123, 125, 127). This is the case for other GPCRs belonging to the rhodopsin/β-adrenergic-like family. As shown by recent crystal structures of GPCRs-coupled with G proteins, the majority of LHCGR CAMs would disrupt this essential TMD3-TMD6 inter-helical stabilizing bridge. That would enable flexibility for the opening of an intracellular crevice *between the IL2 and IL3 and TMD helix 3 and helix 6*, which in turn would allow exposure of key residues potentially involved in G_s_ and G_i_ activation, (99–106). Integrity of the TMD helix 3-helix 6 salt bridge as a requisite for keeping the inactive conformation of glycoprotein hormone receptors is further emphasized by experimental evidence. For example, D567G/N and D619G mutations lead to constitutive activation of the FSH and TSH receptors, respectively (128–133). In addition, *in silico* studies on a number of laboratory manufactured CAM FSHRs harboring mutations at residues 401, 580, 545, and 460 (Figure 1) are known to provoke constitutive activation of the LHCGR (127). It is also noteworthy that the majority of naturally occurring CAMs in the LHCG and TSH receptors are located at the TMD helix 6, which again underlines the importance of this particular helix on G protein coupling and signal transduction.

In contrast to the LHCGR or TSHR, gain-of-function mutations in the 7TMD of the FSHR leading to constitutive activation are relatively rare (Figure 1) despite the relatively high homology between their 7TMD [reviewed in (134)]. This observation suggests a higher stability of the FSHR 7TMD in the inactive state compared with those of other glycoprotein hormone receptors (135). Nevertheless, it is important to keep in mind that CAMs of the FSHR are actually difficult to detect in the clinic because they usually do not exhibit severe phenotypes (136). In fact, mutations leading to ligand-independent activation of LHCGR show low constitutive activity when introduced into the FSHR (127, 135), despite strong promiscuous activation by hCG and TSH (127). Promiscuous activation also has been observed in three out of six naturally occurring FSHR CAMs (134), suggesting a close link between constitutive activation of this receptor and ligand promiscuity, an association not always observed in the other related receptors (91, 130). Partial activation of the FSHR apparently facilitates relaxing the inhibitory constraints of the 7TMD, making the receptor prone to full activation by related ligands when present at high concentrations.

## FSHR domains AND signal transduction

As described above, binding of agonist to the FSHR provokes conformational changes in the receptor molecule, that are transmitted through the 7TMD to the intracellular domains, where coupling to effectors, interaction with adapter proteins, and triggering of downstream intracellular signaling takes place. As in other GPCRs, the intracellular domains of the glycoprotein hormone receptors are extensions of the TMDs, that participate in downstream effector activation. Accordingly, conformational changes in the 7TMD helices lead to activation of G proteins and other interacting proteins involved in signaling, desensitization and internalization of the receptor (15, 56, 58, 122, 137–142) (Figure 1C). In addition to activation of the canonical G_s_/adenylyl ciclase(cAMP/protein kinase A (PKA) pathway, the FSHR also activates signaling cascades involved in a variety of cellular processes, including proliferation and/or differentiation, functional selectivity and differential gene expression [reviewed in (143)]. Some of the motifs involved in these complex signaling networks are shown in Figure 1. For example interaction of the FSHR with the adaptor protein containing pleckstrin homology domain, phosphotyrosine binding domain, and leucine zipper motif (APPL), has been mapped to the IL-1, specifically to K393, L394, and F399 (144, 145). The adapter APPL1 may regulate signal specificity and trafficking through the interaction with PI3K and Akt, which is followed by FOXO1a phosphorylation, leading to abrogation of apoptosis (145); in addition, this adaptor is also involved in FSHR-mediated Ca^2+^ signaling and other functions (84, 146). Meanwhile, association of the FSHR with the 14-3-3τ protein has been mapped to the IL2, overlapping the above mentioned ERW motif (138, 147); 14-3-3 proteins are involved in several cell processes and play an important role in modulating signaling pathways through interacting with activated signaling proteins (148). Mutagenesis studies also have identified other residues in this loop, such as Leu477, that are important for maintaining the receptor in an inactive conformation (142), and it has been suggested that this particular loop may function as a conformational switch to evoke G protein activation, as reported for the LHCGR (58, 149, 150). Sequences in IL3 have been identified that are involved in signal transduction, including the reverse BB*XX*B motif in the juxtamembrane region of this loop (56, 151, 152). Replacement of R573 with cysteine does not affect PM expression or binding to agonist yet signaling mediated by G_s_ is severely impaired (59).

The C-tail exhibits a putative class B S/T cluster closely related with receptor phosphorylation by G protein-coupled receptor kinases (GRKs) and β-arrestin recruitment, which are scaffold intermediates involved not only in receptor desensitization, internalization, and recycling, but also in G_s_-independent ERK1/2-mediated signaling (see below) (153–156).

The C-tail of the FSHR also exhibits an aspargine residue at position 680, which is the site for the expression of the most common functional variant (N680S) of the WT FSHR resulting from a single nucleotide polymorfism (SNP) in the *FSHR* and that exists in strong linkage disequilibrium with the amino acid residue in position 307 (T307A) at the ECD (157). Expression of the S680S FSHR variant *in vivo* has been associated with variations in the sensitivity of the FSHR to its cognate ligand (158, 159), whereas *in vitro* this variant exhibited attenuated intracellular signaling kinetics, enhanced β-arresting recruitment and ligand-stimulated internalization, and decreased CREB-dependent gene transcription and nuclear PKA activation (160, 161). The functional abnormalities of the S680S FSHR variant might be responsible for the altered response to exogenous FSH administration presented by women bearing the homozygous state as well as for the lower pregnancy rates observed in some particular populations (162).

Potentially important domains at the 7TMD and ILs involved in receptor-G protein association have been described in the preceding section.

## FSHR domains involved in internalization AND post-endocytic processing

G protein-coupled receptor interaction with agonist at the PM triggers downward trafficking of the receptor, which occurs through a series of well-known distinct processes. These include: a. phosphorylation and β-arrestin recruitment, which by interacting with clathrin and the clathrin adaptor AP2 promote receptor internalization into endosomes, and b. either targeting of the receptor to the lysosomes and/or proteasomes or recycling of the receptor back to the PM. Hence, the balance between trafficking from the site of synthesis (the ER) to the PM and the endocytosis-recycling/degradation pathway is what defines the final density of receptor protein available to agonist and required to evoke a biological response. Recently, FSHR was identified in very early endosomes during its post-endocytic sorting, rather than to early endosomes as in most GPCRs; apparently, sorting to very early endosomes represents an important mechanism subserving receptor recycling, where PKA-phosphorylated APPL1 present in this particular endosomal compartment plays an essential role (163–165). In addition to phosphorylation by PKA and PKC (both second messenger-dependent kinases), FSHR is phosphorylated by GRKs 2, 3, 5, and 6 (153, 155, 166, 167). Although both PKA and PKC participate in agonist-dependent and -independent desensitization (homologous and heterologous desensitization, respectively) of the FSHR, phosphorylation mediated by GRK results in more complex effects, including homologous desensitization, regulation of β-arrestin recruitment, internalization, and G protein-independent signaling (153). As described in the previous section, a cluster of five serine and threonine residues has been identified in the C-tail of the FSHR as target for phosphorylation by GRKs (153). β-arrestins associated with the GRK2- or GRK5/6-phosphorylated, agonist-occupied FSHR, apparently extert distinct intracellular functions: the FSHR phosphorylated by GRK2 predominates in the β-arrestin-stimulated desensitization process, while phosphorylation by GRK5- and GRK6- is necessary for β-arrestin-mediated MAPK-ERK1/2 activation (153, 154, 168).

β-arrestin recruitment to GRK-phosphorylated FSHR is a well-recognized process leading to receptor internalization (153, 167, 169). In the case of the LHCGR this effect is rather mediated by the interaction with ADP ribosylation factor nucleotide-binding site opener (ARNO), which is an exchange factor for ADP ribosylation factor 6 (ARF6) that recruits β-arrestins when bound to GTP (170, 171). In contrast with the LHCGR (in which only 30% of the internalized receptor recycles back to the PM), most of the internalized FSHR is recycled back to the cell surface (166, 172). Palmitoylation plays and important role in determining the post-endocytic fate (degradation vs. recycling) of gonadotropin receptors (17, 18, 173–175). The importance of this S-acylation in internalization and post-endocytic processing of GPCRs varies depending on the particular receptor. In contrast to the LHCGR in which prevention of palmitoylation by site-directed mutagenesis increased the rate of agonist-stimulated internalization (174), abrogation of palmitoylation of the C-tail cysteine residues (cysteines 644, 646, and 672, Figure 1C) at the FSHR did not affect the dynamics of internalization of the hormone/FSHR complex (172). Nevertheless, in both unpalmitoylated receptors, recycling to the cell surface was impaired and the fraction of receptor/hormone complex submitted to degradation via the proteasome/lysosome pathway was increased (17, 174). Further, studies in HEK293 cells showed that in the non-palmitoylated FSHR degradation through proteasomes predominated over that mediated by lysosomes, as revealed by experiments in which proteosomal but not lysosomal degradation was inhibited (17). In fact, the FSHR is ubiquitinated in IL3 (Figure 1C) and proteosomal inhibitors increase cell surface residency of this receptor (17, 176). Thus in both gonadotropin receptors, S-acylation plays an important role in postendocytic processing.

In addition to palmitoylation, postendocytic trafficking also may be influenced by specific amino acid residues present in the C-tail of the FSHR. Similar to the LHCGR, truncations involving the last eight residues of the FSHR resulted in re-routing of a substantial amount of the internalized FSH-FSHR complex to the degradation pathway (166).

## Conclusions

This review summarizes the information available on the relationship between structure and function of the FSHR. Although a substantial amount of information on this particular receptor structure-activity relationship has emerged during the last decade, there are still several issues that remain to be resolved, including elucidation of the entire crystal structure of the receptor including the 7TMD. This critical step will unambiguously and more precisely identify those residues and domains within the 7TMD and intracellular domains involved in receptor activation, FSHR-FSHR and FSHR/LHCGR association, and interaction with the array of proteins involved in intracellular signaling, and also in specific binding of allosteric modulators, the latter with important implications in the clinical arena.

Since there is no firm structural data on whether reported extragonadal FSHRs are variants of the canonical FSHR structure, particularly the FSHRs represented to be in bone, adipose tissue and malignant tumors (33, 177, 178), a more precise identification of such structural features might allow the design of highly specific therapeutic strategies, which block putative deleterious FSH effects on these particular tissues. In this vein, application of novel imaging techniques (179) may be useful to critically evaluate whether expression levels of FSHR in those extragonadal tissues are sufficient to incur these deleterious effects or whether their density changes as the menopausal status progresses. Without any doubt, crystals of gonadotropin receptors also will aid to clarify many aspects on extragonadal FSHRs function that may be translated in the near-term to human therapeutics.

Finally, another interesting issue concerns to the altered response of the S680S FSHR variant to the FSH stimulus. In this regard, two novel therapeutic FSH compounds produced by human cell lines have emerged; comparatively, these preparations differ somehow in glycosylation pattern and apparently exhibit a more favorable pharmacodynamic profile than the recombinant preparations synthesized by non-human cell lines (180–182). Those novel FSH preparations might be more advantageous than the widely used non-human cell-derived FSH compounds in women bearing the less favorable S680S FSHR variant. Nonetheless, more detailed data on the structural and biochemical features of these human cell-derived FSH preparations as well as on their binding dynamics at the FSHR and, more importantly, their effects on intracellular signaling, still are necessary before considering these new FSH formulations as a worthy option for these women.

## Author contributions

AU-A, TZ, EJ-V, RG-S, and JD wrote the manuscript. AU-A and JD reviewed and edited the final version.

### Conflict of interest statement

The authors declare that the research was conducted in the absence of any commercial or financial relationships that could be construed as a potential conflict of interest.

## Footnotes

^1^Amino acid numbering according to the Ballesteros and Weinstein nomenclature, in which the first number denotes the helix (1–7) and the second the residue position relative to the most conserved position, which is assigned the number 50. [see Ballesteros and Weinstein (108)].
